# Fractional B-Spline Wavelets and U-Net Architecture for Robust and Reliable Vehicle Detection in Snowy Conditions

**DOI:** 10.3390/s24123938

**Published:** 2024-06-18

**Authors:** Hamam Mokayed, Christián Ulehla, Elda Shurdhaj, Amirhossein Nayebiastaneh, Lama Alkhaled, Olle Hagner, Yan Chai Hum

**Affiliations:** 1 Electrical and Space Engineering, Luleå University of Technology, 97187 Luleå, Sweden; chrule-2@student.ltu.se (C.U.); eldshu-2@student.ltu.se (E.S.); aminay-2@student.ltu.se (A.N.); lama.alkhaled@ltu.se (L.A.); 2 Smartplanes, Jävre, 94494 Piteå Municipality, Sweden; olle.hagner@smartplanes.se; 3 Mechatronics and Biomedical Engineering, Universiti Tunku Abdul Rahman, Jalan Sungai Long, Bandar Sungai Long, Kajang 43000, Selangor, Malaysia; humyc@utar.edu.my

**Keywords:** vehicle detection, fractional B-spline, U-Net, harsh weathers

## Abstract

This paper addresses the critical need for advanced real-time vehicle detection methodologies in Vehicle Intelligence Systems (VIS), especially in the context of using Unmanned Aerial Vehicles (UAVs) for data acquisition in severe weather conditions, such as heavy snowfall typical of the Nordic region. Traditional vehicle detection techniques, which often rely on custom-engineered features and deterministic algorithms, fall short in adapting to diverse environmental challenges, leading to a demand for more precise and sophisticated methods. The limitations of current architectures, particularly when deployed in real-time on edge devices with restricted computational capabilities, are highlighted as significant hurdles in the development of efficient vehicle detection systems. To bridge this gap, our research focuses on the formulation of an innovative approach that combines the fractional B-spline wavelet transform with a tailored U-Net architecture, operational on a Raspberry Pi 4. This method aims to enhance vehicle detection and localization by leveraging the unique attributes of the NVD dataset, which comprises drone-captured imagery under the harsh winter conditions of northern Sweden. The dataset, featuring 8450 annotated frames with 26,313 vehicles, serves as the foundation for evaluating the proposed technique. The comparative analysis of the proposed method against state-of-the-art detectors, such as YOLO and Faster RCNN, in both accuracy and efficiency on constrained devices, emphasizes the capability of our method to balance the trade-off between speed and accuracy, thereby broadening its utility across various domains.

## 1. Introduction

Vehicle detection stands as a crucial element within most Vehicle Intelligence Systems (VIS), which are typically employed to ensure safety, optimize traffic flow, and enable autonomous driving. Traditional approaches to vehicle detection often relied on Custom-engineered features and rule-based algorithms, resulting in a constrained capacity to adjust to different environmental scenarios. Furthermore, with the escalation in the complexity of real-life situations, there emerged a clear need for detection methodologies that were both more precise and advanced [1]. While complicated architectures may enhance detection precision, they introduce additional hurdles, particularly in the context of real-time applications operating on devices with limited capabilities. In many critical transportation solutions where drones are used for data acquisition and processing, a range of difficulties arises. Among these, issues concerning images captured by drones are notable, including oblique angles, non-uniform illumination, degradation, blurring, occlusion, and reduced visibility [2]. Concurrently, the necessity for on-the-fly processing as the drone captures data imposes constraints on the available computational resources [3]. The lack of sufficient data and research on vehicle detection using drones in snowy conditions is the major drive of this work [4]. Many researchers have concentrated on improving the detection of various objects, such as lanes and traffic lights, in different environments. However, it remains uncertain how these advancements will perform in the context of our specific vehicle detection challenges [5,6]. The goal is to shed light on vehicle detection challenges in snowy conditions using drones and offer valuable insights to improve the accuracy and reliability of object detection systems in adverse weather scenarios. Furthermore, this investigation seeks to assess the effectiveness of the proposed methodologies on edge computing devices and to conduct comparative analyses of both the performance and accuracy against state-of-the-art detection frameworks such as YOLO and Faster RCNN. At the inception of this study, a thorough review of the recent research was conducted, focusing on research that addresses vehicle detection on edge devices under severe weather conditions. This search aimed to establish a solid foundation for the current work by identifying gaps in the existing body of knowledge and confirming the necessity of further exploration in this area. The investigation revealed a significant lack of studies that specifically tackle the challenges associated with real-time vehicle detection using edge computing in adverse weather scenarios, such as heavy snow. This finding not only underscored the relevance and urgency of the present research but also highlighted the potential for contributing novel insights and methodologies to the field of intelligent transportation systems and computer vision, particularly in enhancing the robustness and efficiency of vehicle detection technologies in less-than-ideal environmental conditions. Table 1 summarizes the findings from our inquiry into the previously implemented models by researchers, the specific customizations applied, the specifications of the edge device employed, and the weather conditions under which the system was tested.

These above-mentioned studies collectively push the boundaries of object detection, providing tailored solutions to meet distinct needs within various application domains. However, these advancements are not without their challenges. Common obstacles across these studies include navigating the trade-off between detection speed and accuracy, ensuring consistent performance across different environmental conditions, and managing the computational demands of sophisticated models without sacrificing their effectiveness. Such challenges underscore the inherent complexities in object detection and the continuous need for innovative solutions and optimizations [16].

## 2. Proposed Method

The advancement of vehicle detection methods, especially with UAV images, highlights a specific instance where deep learning has played significant improvements. Nevertheless, the complexity and diversity of features in aerial images captured in severe weather conditions demand further enhancements. In response to this requirement, the proposed study proposes a technique that utilizes the fractional B-spline wavelet transform together with a customized U-Net architecture, implemented on a Raspberry Pi 4 Model B. This strategy is specifically designed to enhance vehicle detection and localization, with an emphasis on evaluating its efficacy using the NVD dataset. The NVD dataset [4], accessible at https://nvd.ltu-ai.dev/ (accessed on 12 June 2024), and a full explanation of the data extraction process can be found at [4]. NVD was compiled amidst the harsh snowy winter conditions of northern Sweden. It encompasses a collection of images taken from heights varying between 120 and 250 m, including 8450 frames that have been annotated to highlight 26,313 vehicles, alongside approximately 10 h of video content that awaits annotation. This dataset is characterized by its variation in video resolutions and frame rates, as well as differences in Ground Sample Distance (GSD) measurements, providing a comprehensive portrayal of vehicles under the demanding winter weather typical of the Nordic region.

### 2.1. Fractional B-Spline Wavelet Transform

A two-dimensional fractional B-spline wavelet transform was applied to extract relevant features from aerial images. The Fractional B-spline wavelet Transform is an advanced mathematical tool that extends the traditional B-spline wavelet transform by incorporating the concept of fractional calculus. Fractional calculus allows for operations that can be applied at any real or complex order, providing more flexible and precise analysis of data, especially when dealing with complex patterns or irregularities. This extension allows for a more flexible manipulation of the wavelet functions, enabling the extraction of features with varying degrees of smoothness and detail from an image. The “fractional” aspect refers to the use of non-integer derivatives, which provide a richer set of parameters to adjust the wavelet functions, thereby offering more control over the feature extraction process. The fractional nature allows for better detection of edges and boundaries in snowy conditions where the edges of vehicles can become blurred or indistinct. This transform also can analyze images at multiple scales, this is particularly useful for drone imagery, where vehicles can appear at various sizes and orientations. The implementation involves applying wavelet filters separately to vertical and horizontal dimensions. Usually, the wavelet transform involves decomposing a signal into shifted and scaled versions of a base wavelet function. In the fractional domain, this is extended by employing fractional B-spline functions as the base wavelets. The transform coefficients at a scale α and shift *b* for a signal *f*(*t*) using a fractional wavelet $\Psi^{\alpha }\left(t\right)$ (derived from the fractional B-spline functions) can be defined as in Equation (1)

(1) $$W_{f}^{\alpha }\left(a,b\right)=\frac{1}{\sqrt{|\alpha |}}\int f\left(t\right)\bar{\Psi^{\alpha }(\frac{t-b}{a}})dt$$

where $\bar{\Psi^{\alpha }(\frac{t-b}{a}})$ denotes the complex conjugate of $\Psi^{\alpha }\left(\frac{t-b}{a}\right)$.

**Figure 1:**
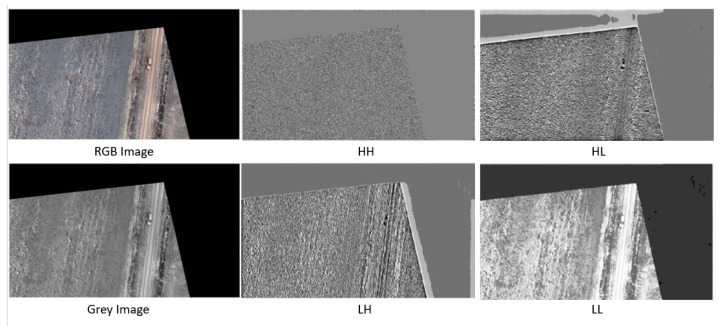
Sample of implementing the Fractional B-Spline Wavelet Transform over NVD dataset.

The resulting High-Low (HL) and Low-High (LH) channels were found to contain valuable information for car detection as shown in Figure 1.

### 2.2. Integration with U-Net

**Figure 2:**
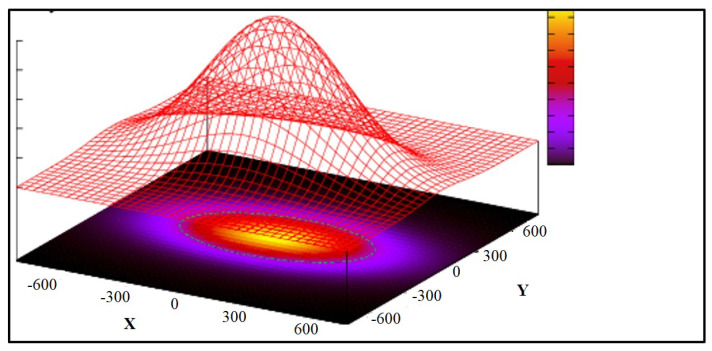
Illustration of gaussian elliptical function over heatmap.

**Figure 3:**
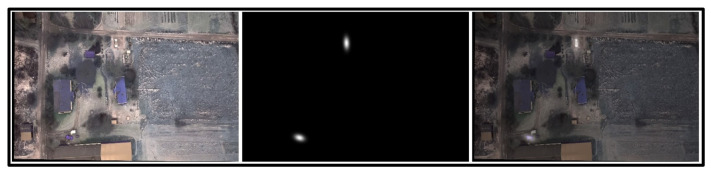
From left to right: original image, produced heatmaps, overlay of both.

The fractional B-spline wavelet transform was implemented to utilize the provided two-dimensional fractional spline wavelet transform. The transform was applied to each channel of the input image, producing 4 different channels. Only LH and HL channels were then resized and concatenated with the output of the first convolutional layer before being input into the second convolutional layer of the U-Net architecture. The U-Net architecture, named CarLocalizationCNN, was employed for its effectiveness in semantic segmentation tasks. Notably, we modified the U-Net by incorporating the transformed channels into its second convolutional layer. The first layer output channels and the fractional B-spline transformed channels were concatenated and used as input to the second convolutional layer of the network. This approach aims to enhance the network’s ability to discern subtle features related to car presence. Skip connections, present in the original U-Net structure, were intentionally omitted to streamline the architecture for heatmap generation without accurate car boundary delineation. The output of the CarLocalizationCNN model is designed as a heatmap. The final layer of the network produces a heatmap highlighting potential car locations in the input image. This heatmap serves as a valuable tool for visualizing and interpreting the network’s car detection predictions. The generation of these heatmaps is done through the application of a Gaussian elliptical function. The Gaussian function has been selected to facilitate a gradual increase in pixel intensity (a proxy for the likelihood of the presence of a vehicle) as one moves toward the central region of the car. This feature ensures smoothness for the gradient descent during the testing process [17]. Considering the rectangular shape of cars, we opted for an elliptical function, which entails setting one dimension of the Gaussian function with a higher sigma value compared to the other. This approach allows us to better represent the vehicles’ shape in the analysis. Furthermore, we rotated this elliptical Gaussian function using values derived from the original annotations to ensure an optimal fit to the vehicle’s orientation as shown in both below figures (Figure 2 and Figure 3).

**Figure 4:**
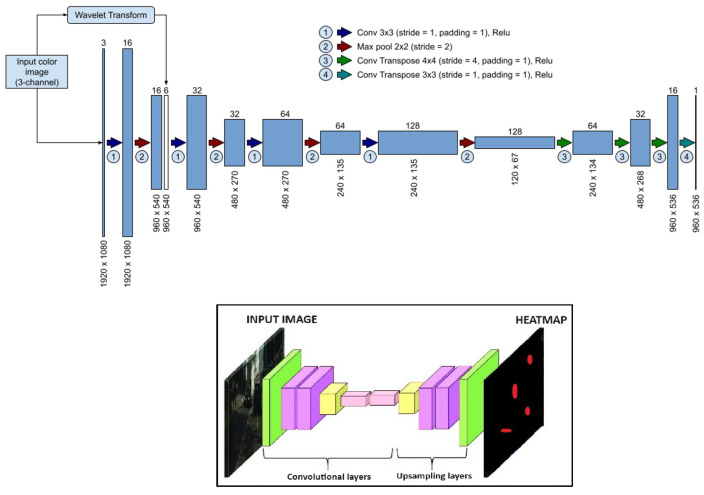
CarLocalizationCNN architecture (CNN)-based U-Net model).

The CarLocalizationCNN model consists of convolutional and up-sampling layers. The convolutional layers capture hierarchical features, while the up-sampling layers restore spatial information as shown in Figure 4. The integration of the fractional B-spline transformed channels into the network is handled seamlessly within the architecture, culminating in a heatmap highlighting potential car locations.

## 3. Dataset

**Figure 5:**
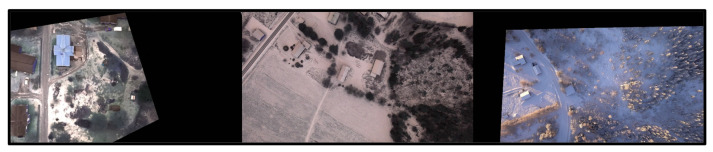
Samples of NVD frames.

Models have been trained and assessed using frames taken from videos that vary in several aspects, including height, snow coverage, cloud coverage, and Ground Sample Distance (GSD) pixel dimensions. The video information and samples of the extracted frame are shown in Table 2 and Table 3, and Figure 5.

## 4. Testing and Evaluation

The outcomes of the proposed model compared against three detectors commonly utilized in are widely used in both academic research and industrial applications.

- YOLOv5s.
- YOLOv8s.
- Faster R-CNN.

### 4.1. Evaluation Metrics and Benchmarking

**Figure 6:**
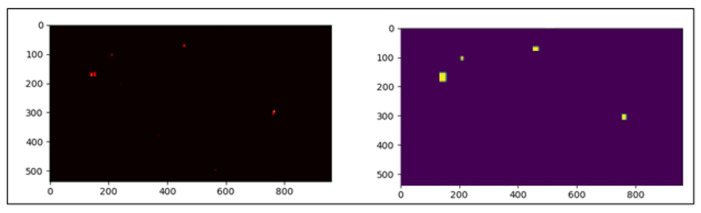
Conversion of the heatmap to bounding boxes.

The main evaluation metrics that the models will be compared with are mean Average Precision (mAP) and Inference Time. Since the output of the proposed model is a heatmap, the heatmap was converted to a reflective bounding box by thresholding and grouping the heatmap pixels to be represented as a bounding box in Figure 6.

After we have extracted these bounding boxes representing car predictions, we need to compare them with actual annotated bounding boxes. For this comparison, we employ an adapted version of the Intersection over Union (IoU) metric, which essentially calculates the proportion of the overlapping area between two bounding boxes relative to their combined area, ensuring the overlapped section is accounted for only once (Union).

- When a pair of bounding boxes (one from the predictions and one from the ground truth) achieves an IoU exceeding a predefined threshold, we classify the prediction as accurate, or a True Positive.
- Should a predicted bounding box fail to meet the IoU threshold with any ground-truth bounding boxes, we categorize the prediction as a False Positive.
- Conversely, if a ground-truth bounding box does not reach the IoU threshold with any predicted bounding boxes, we label the prediction as a False Negative.

### 4.2. Experimental Results

**Figure 7:**
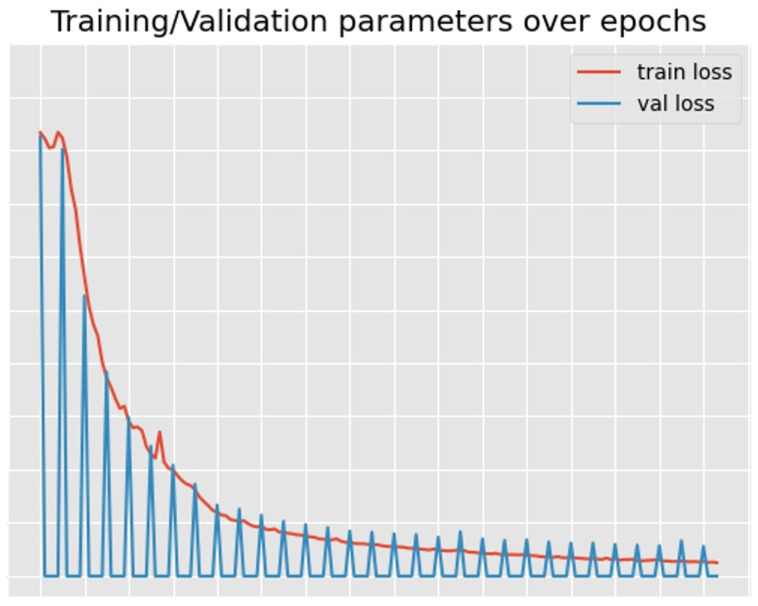
Loss of the proposed model.

The training of the mentioned models was operated using a PC specified in Table 4 with a training/validation loss of the proposed model during the training as shown in Figure 7. Meanwhile, the evaluation was conducted on Raspberry Pi 4 Model B.

**Figure 8:**
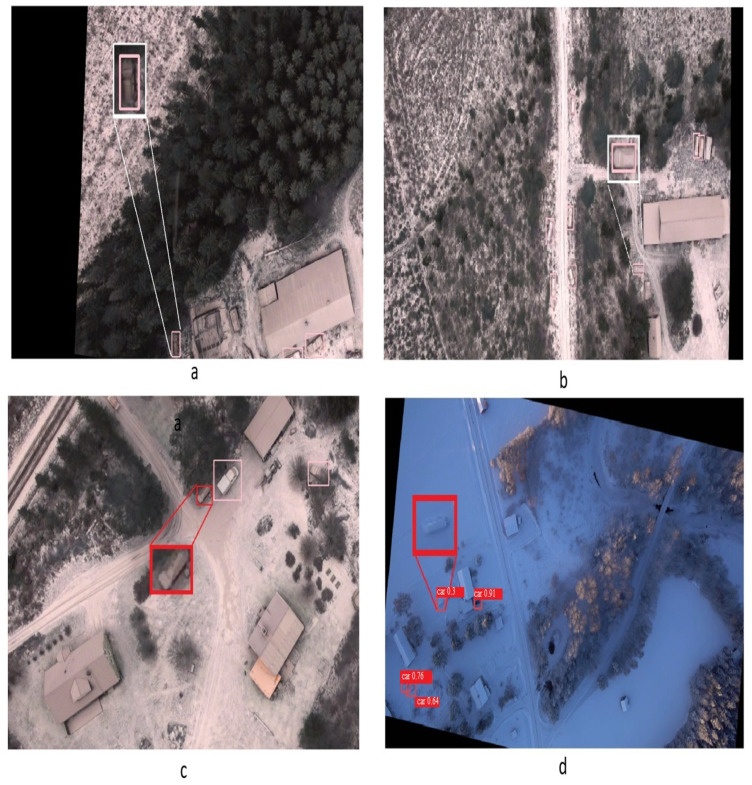
Detection comparisons among the different detectors. (**a**) detected by calLocalization only, (**b**) carLocalization and YOLO8s_aug, (**c**) not detected in all, (**d**) detected by YOLO8s_aug but carLocalization.

The core aim of the experimental outcomes is to assess the model’s performance through two essential metrics: mean average precision (mAP50) for accuracy, and inference time for evaluating efficiency. mAP50 measures how well the model predicts and localizes objects, indicating its accuracy, while inference time assesses the model’s speed in processing images, reflecting its practical utility in real-time applications. These metrics collectively provide a concise evaluation of the model’s overall effectiveness and applicability as shown in Table 5 and Table 6, and Figure 8.

The proposed carLocalizationCNN model demonstrates an enhancement in recall, mAP50, and mAP50-90 metrics when contrasted with YOLOv8s, YOLOv5s, YOLOv5s_aug*, YOLOv8s_aug*, and Faster R-CNN (FRCNN). These improvements highlight the model’s enhanced ability to correctly identify and localize vehicles across a range of conditions and overlaps, thus offering a more robust solution for vehicle detection tasks. However, it is worth mentioning that YOLOv8s_aug outperforms the proposed model in terms of precision. This indicates that while the carLocalizationCNN model is adept at reducing false negatives and improving detection coverage, YOLOv8s maintains a higher accuracy in predicting true positive detections, minimizing false positives within its identifications.

On the efficiency, The model we’ve developed demonstrates enhanced efficiency in its inference capabilities when compared with several established models. Specifically, it processes images approximately 1.83 times quicker than YOLOv5s, signifying a notable speed improvement, which can be particularly beneficial in scenarios demanding rapid data processing. Compared to YOLOv5s, the proposed model exhibits a modest speed increment of 1.25%, which, while smaller, still reflects an advancement in processing efficiency. While the model outperforms Faster R-CNN (FRCNN) by a factor of 6.7, indicating a significant leap in inference speed. This good acceleration in processing times could enhance the applicability of the model in real-time applications like the case that we are discussing using drones for decision-making. The improvements underscore the model’s potential in balancing the trade-off between speed and accuracy, thereby broadening its utility across various domains.

## 5. Ethical Considerations

- Accuracy: Emphasis was placed on the high-quality annotations of the dataset, which is specific to snowy conditions in the Nordic region, acknowledging potential limitations in generalizability and the importance of accurate data for training algorithms.
- Transparency: The methodology for data collection and model training is thoroughly documented, promoting scrutiny and validation by the scientific community. The use of deep learning for vehicle detection is well-explained, with intentions to share findings and ensure the algorithms perform as expected without unintended behaviors. The dataset used is publicly available.
- Privacy: The aerial data collection minimizes privacy risks by focusing on vehicle tops, excluding identifiable details like license plates or human faces, ensuring anonymity and compliance with privacy standards.
- Fairness and Bias: The dataset encompasses a diverse range of vehicles under snowy conditions to mitigate bias. Careful dataset splitting ensures balanced training and testing sets, allowing for an equitable assessment of the model’s performance in snow-covered environments.

## 6. Conclusions and Future Work

In conclusion, this study represents a step forward in the domain of Vehicle Intelligence Systems (VIS), especially for real-time vehicle detection in adverse weather conditions using Unmanned Aerial Vehicles (UAVs). The proposed method addresses some of these challenges presented by heavy snowfall, common in Nordic regions. The carLocalizationCNN model developed here adds an enhancement to traditional detection methods like YOLO and Faster R-CNN in key metrics such as recall, mAP50, and mAP50-90. Moreover, the model exhibits improvements in inference speed, making it highly suitable for time-sensitive applications like UAV-based surveillance in snowy environments. The research also seeks to highlight the existing research gap highlighted by the NVD dataset. There remains a substantial need for further investigation to enhance vehicle detection capabilities in such harsh weather conditions. The extensive evaluation using the NVD dataset lays the groundwork for future research in VIS, particularly in optimizing performance for edge devices operating in challenging environments.

Future work should focus on developing detection algorithms specifically tailored for snowy conditions, accounting for unique visual challenges such as reduced contrast, varying snow textures, and occlusions caused by snow accumulation. Techniques like multi-scale feature extraction and context-aware detection can be explored to enhance robustness. Additionally, the use of more advnaced signal processing techniques can offer improved flexibility in modeling the irregular and complex shapes of snow-covered vehicles, potentially leading to more accurate detections. Research can explore optimizing the parameters of fractional splines to balance computational efficiency and detection accuracy.

## Figures and Tables

**Table 1:** Applied search criteria over available vehicle dataset.

Paper	Model	Edge Device	Dataset/Weather Condition
Li et al., 2023 [7]	YOLOv5s, YOLOv5	CPU Intel(R) Xeon(R) Platinum 8358P CPU @ 2.60 GHz, GPU: RTX A5000-24 GB.	VisDrone2019-DET No specific weather conditions.
Bulut et al., 2023 [8]	YOLOv5, YOLOv7, YOLOv6	NVIDIA Jetson Nano	No specific weather conditions.
Liu et al., 2023 [9]	improved version of the YOLOv5 called YOLO-Extract.	X	DOTA dataset. No specific weather conditions.
Huang et al., 2023 [10]	YOLOv4	NVIDIA Jetson Nano	No specific weather conditions.
Mokayed et al., 2024 [11]	YOLOv5s, YOLOv8s, SSD, FRCNN	X	NVD dataset with severe snowy conditions
John et al., 2023 [12]	YOLOv5 and YOLOv7, with YOLOv7 being the primary model	X	VEDAI dataset
Javid et al., 2024 [13]	(CNN)-based U-Net model	X	DLR3K and Vedai datasets.
Tanasa et al., 2023 [14]	U-Net	NVIDIA Quadro RTX 4000 GPUs with 8 GB of memory, and NVIDIA Pascal GPU-based TX2 edge devices with 8 GB of memory.	No specific weather conditions.
Mokayed et al., 2021 [15]	DCT-PCM with conventional CNN	NUC intel i7 processor without GPU.	Mimos dataset, no specific weather conditions.

**Table 2:** Training dataset, 3 videos with 6056 annotated frames include 18,976 vehicles.

Video	Altitude	Snow Cover	Cloud Cover	fps	GSD
Asjo 01	130–200 m	minimal (0–1 cm)	overcast	5	11.5–17.8 cm
Bjenberg	250 m	Fresh (1–2 cm)	light	25	22.2 cm
Asjo 01 HD	250 m	Fresh (5–10 cm)	clear	5	20.2 cm

**Table 3:** Testing dataset, 2 videos with 2394 annotated frames include 7337 vehicles.

Video	Altitude	Snow Cover	Cloud Cover	fps	GSD
Bjenberg 02	250 m	Fresh (5–10 cm)	clear	5	11.1cm
Nyland-01	150 m	Minimal (0–1 cm)	Dense	5	11.5–17.8 cm

**Table 4:** Specification of the PC used for training.

Part	Specification
Processor	Intel i9-9900K @ 3.6 GHz
RAM	64 GB (3600 MHz) DDR4 CL16
Graphic Card	Nvidia Geforce RTX 3800Ti 12 GB (Cuda 11.1)

**Table 5:** Accuracy comparison between STOA detectors and the proposed model.

Model	Precision	Recall	mAP50	mAP50-95
YOLOv5s	54.2%	33.7%	47.3%	30.5%
YOLOv8s	65.8%	22.4%	45.1%	29.8%
YOLOv5s_aug*	70.6%	48.2%	56.0%	24.1%
YOLOv58s_aug*	77.1%	34.6%	50.7%	24.1%
Faster RCNN (FPN3x)	-	-	46.2%	-
CarLocalizationCNN	74.7%	54.8%	60.4%	38.5%

*_aug means with augmentation, refer to Mokayed et al., 2024 [4].

**Table 6:** Efficiency comparison between STOA detectors and the proposed model.

Model	Pre-Process (ms)	Inference (ms)	Post-Process (ms)	Total (ms)
YOLOv5s	52.6	17,731.7	2.6	17,786.9
YOLOv8s	55.5	12,137.4	3.7	12,196.6
Faster RCNN (FPN3x)	-	62,715.87	-	62,715.87
CarLocalizationCNN	0.032	9348.062	361.172	9709.266

## Data Availability

Data can be accessed at https://nvd.ltu-ai.dev/ accessed on 10 June 2024.

## References

[1] Xu Y., Liu X., Cao X., Huang C., Liu E., Qian S., Liu X., Wu Y., Dong F., Qiu C.W. (2021). Artificial intelligence: A powerful paradigm for scientific research. Innovation.

[2] Tahir N.U.A., Zhang Z., Asim M., Chen J., ELAffendi M. (2024). Object Detection in Autonomous Vehicles under Adverse Weather: A Review of Traditional and Deep Learning Approaches. Algorithms.

[3] Chen C., Wang C., Liu B., He C., Cong L., Wan S. (2023). Edge intelligence empowered vehicle detection and image segmentation for autonomous vehicles. IEEE Trans. Intell. Transp. Syst..

[4] Mokayed H., Nayebiastaneh A., De K., Sozos S., Hagner O., Backe B. Nordic Vehicle Dataset (NVD): Performance of vehicle detectors using newly captured NVD from UAV in different snowy weather conditions. Proceedings of the IEEE/CVF Conference on Computer Vision and Pattern Recognition.

[5] Lee S.H., Lee S.H. (2024). U-Net-Based Learning Using Enhanced Lane Detection with Directional Lane Attention Maps for Various Driving Environments. Mathematics.

[6] Chen B., Fan X. (2024). MSGC-YOLO: An Improved Lightweight Traffic Sign Detection Model under Snow Conditions. Mathematics.

[7] Li S., Yang X., Lin X., Zhang Y., Wu J. (2023). Real-time vehicle detection from UAV aerial images based on improved YOLOv5. Sensors.

[8] Bulut A., Ozdemir F., Bostanci Y.S., Soyturk M. Performance Evaluation of Recent Object Detection Models for Traffic Safety Applications on Edge. Proceedings of the 2023 5th International Conference on Image Processing and Machine Vision.

[9] Liu Z., Gao Y., Du Q., Chen M., Lv W. (2023). YOLO-extract: Improved YOLOv5 for aircraft object detection in remote sensing images. IEEE Access.

[10] Huang F., Chen S., Wang Q., Chen Y., Zhang D. (2023). Using deep learning in an embedded system for real-time target detection based on images from an unmanned aerial vehicle: Vehicle detection as a case study. Int. J. Digit. Earth.

[11] Mokayed H., Nayebiastaneh A., Alkhaled L., Sozos S., Hagner O., Backe B. Challenging YOLO and Faster RCNN in Snowy Conditions: UAV Nordic Vehicle Dataset (NVD) as an Example. Proceedings of the 2024 2nd International Conference on Unmanned Vehicle Systems-Oman (UVS).

[12] John S.M., Kareem F.A., Paul S.G., Gafur A., Al Mansoori S., Panthakkan A. Enhanced YOLOv7 Model for Accurate Vehicle Detection from UAV Imagery. Proceedings of the 2023 International Conference on Innovations in Engineering and Technology (ICIET).

[13] Javid I., Ghazali R., Saeed W., Batool T., Al-Wajih E. (2023). CNN with New Spatial Pyramid Pooling and Advanced Filter-Based Techniques: Revolutionizing Traffic Monitoring via Aerial Images. Sustainability.

[14] Tanasa I.Y., Budiarti D.H., Guno Y., Yunata A.S., Wibowo M., Hidayat A., Purnamastuti F.N., Purwanto A., Wicaksono G., Domiri D.D. U-Net Utilization on segmentation of Aerial Captured Images. Proceedings of the 2023 International Conference on Radar, Antenna, Microwave, Electronics, and Telecommunications (ICRAMET).

[15] Mokayed H., Shivakumara P., Woon H.H., Kankanhalli M., Lu T., Pal U. (2021). A new DCT-PCM method for license plate number detection in drone images. Pattern Recognit. Lett..

[16] Chen Z., Yang J., Feng Z., Zhu H. (2024). RailFOD23: A dataset for foreign object detection on railroad transmission lines. Sci. Data.

[17] McKean H., Moll V. (1999). Elliptic Curves: Function Theory, Geometry, Arithmetic.

